# HOME DM-BAT: home-based diabetes-modified behavioral activation treatment for low-income seniors with type 2 diabetes—study protocol for a randomized controlled trial

**DOI:** 10.1186/s13063-021-05744-1

**Published:** 2021-11-08

**Authors:** Leonard E. Egede, Tatiana M. Davidson, Rebecca G. Knapp, Rebekah J. Walker, Joni S. Williams, Clara E. Dismuke, Aprill Z. Dawson

**Affiliations:** 1 Department of Medicine, Division of General Internal Medicine, Medical College of Wisconsin, 8701 Watertown Plank Rd, Milwaukee, WI 53226 USA; 2 Center for Advancing Population Science, Medical College of Wisconsin, 8701 Watertown Plank Rd, Milwaukee, WI 53226 USA; 3 College of Nursing, Medical University of South Carolina, 99 Jonathan Lucas St, MSC 160, Charleston, SC 29425 USA; 4 Department of Public Health Services, College of Medicine, Medical University of South Carolina, 135 Cannon St., Charleston, SC 29425 USA; 5 Health Economics Resource Center, VA Palo Alto Healthcare System, 795 Willow Road (152 MPD), Menlo Park, CA 94025 USA

**Keywords:** Diabetes, Elderly, Seniors, Older adults, Education, Behavioral activation, Skills training

## Abstract

**Background:**

About 13% of African Americans and 13% of Hispanics have diabetes, compared to 8% of non-Hispanic Whites (NHWs). This is more pronounced in the elderly where about 25–30% of those aged 65 and older have diabetes. Studies have found associations between social determinants of health (SDoH) and increased incidence, prevalence, and burden of diabetes; however, few interventions have accounted for the context in which the elderly live by addressing SDoH. Specifically, psychosocial factors (such as cognitive dysfunction, functional impairment, and social isolation) impacting this population may be under-addressed due to numerous medical concerns addressed during the clinical visit. The long-term goal of the project is to identify strategies to improve glycemic control and reduce diabetes complications and mortality in African Americans and Hispanics/Latinos with type 2 diabetes.

**Methods:**

This is a 5-year prospective, randomized clinical trial, which will test the effectiveness of a home-based diabetes-modified behavioral activation treatment for low-income, minority seniors with type 2 diabetes mellitus (T2DM) (HOME DM-BAT). Two hundred, aged 65 and older and with an HbA1c ≥8%, will be randomized into one of two groups: (1) an intervention using in-home, nurse telephone-delivered diabetes education, and behavioral activation or (2) a usual care group using in-home, nurse telephone-delivered, health education/supportive therapy. Participants will be followed for 12 months to ascertain the effect of the intervention on glycemic control, blood pressure, and low-density lipoprotein (LDL) cholesterol. The primary hypothesis is low-income, minority seniors with poorly controlled type 2 diabetes randomized to HOME DM-BAT will have significantly greater improvements in clinical outcomes at 12 months of follow-up compared to usual care.

**Discussion:**

Results from this study will provide important insight into the effectiveness of a home-based diabetes-modified behavioral activation treatment for low-income, minority seniors with uncontrolled type 2 diabetes mellitus and inform strategies to improve glycemic control and reduce diabetes complications in minority elderly with T2DM.

**Trial registration:**

ClinicalTrials.govNCT04203147). Registered on December 18, 2019, with the National Institutes of Health Clinical Trials Registry.

## Administrative Information

**Dissemination Policy:** This trial has been registered with ClinicalTrials.gov. Information will be updated annually and within 30 days of trial completion. Study-related findings will be presented at scientific meetings and published in peer-reviewed, PubMed-indexed journals. Data documentation and de-identified data associated with the study will be shared at the NIDDK Central Repository, an NIH-funded repository. The NIH repository has policies and procedures in place that will provide data access to qualified researchers, fully consistent with NIH data sharing policies and applicable laws and regulations.

## Background

Rates of diabetes are higher in minority groups compared to non-Hispanic whites (NHW), with 13% of African Americans and 13% of Hispanics diagnosed with diabetes compared to 8% of NHWs [1–3]. Minorities consistently show a higher risk of complications, higher mortality rates, poorer self-management, and worse glycemic control than NHWs [4, 5]. Rates of diabetes are also high in the elderly with 25–30% of individuals aged 65 or older diagnosed [3, 6]. According to the Department of Health and Human Services and the Administration on Aging (AoA), the elderly represent 15% of the total US population and this number is expected to nearly double within the next two decades [7]. In elderly populations, disparities are compounded by additional differences in risk factors such as multimorbidity, cognitive decline, and sociodemographic factors [8–11]. In addition, socioeconomic status adds to disparities, highlighting the need for interventions targeted towards low-income minority seniors with diabetes [12].

Social determinants of health include conditions such as availability of resources to meet daily needs, access to educational, economic and job opportunities, access to health care services, availability of community-based resources and opportunities, transportation options, social support, and socioeconomic conditions influence health status [13]. Other social factors that disproportionally impact minorities such as racism, discrimination, and segregation are also associated with poor outcomes for elderly adults with diabetes [10, 14, 15].

Studies have found associations between social determinants of health and increased incidence, prevalence, and burden of diabetes; however, few interventions have accounted for the context in which people live by directly addressing the social determinants of health, especially in the elderly. Due to significant medical concerns to be addressed in the clinical visit, psychosocial factors impacting elderly patients with diabetes such as cognitive dysfunction, complex treatment regimes, advanced disease, functional impairment, and limited social and financial resources may also be under-addressed [16, 17]. Social isolation is also an important psychosocial factor that has been shown to negatively impact health outcomes in the elderly [18, 19]. More importantly, research from our team has shown that mutable psychosocial determinants of health account for about 25% of variance in glycemic control [20, 21].

Behavioral activation is a cognitive behavior therapy, originally developed to address depression, that has shown promise in individuals with chronic medical conditions [22–26]. It is a psychotherapeutic process whereby patients are encouraged to identify and schedule structured and enjoyable activities for behavior change that is likely to improve outcomes such as mood, behaviors, and quality of life [27]. The focus on identifying and increasing the incorporation of activities into a patient’s daily schedule lends behavioral activation to incorporation into treatment for patients with diabetes [23]. In fact, several pilot studies suggest behavioral activation can be effective in improving self-care behaviors and adherence to treatment recommendations for patients with diabetes [24–26].

Many experts have emphasized the importance of evidence-based treatments (EBTs) accounting for a patient’s cultural contexts and values, including the integration of cultural values, collaboration with individuals familiar with patients’ culture, provision of extra services to increase patient retention, and cultural sensitivity training for professional staff [28–30]. Application of cultural sensitivity frameworks has been successful in maintaining treatment fidelity to the treatment protocol, in participant retention and engagement, and in positive outcomes among Latina/os and African Americans suffering from chronic health (e.g., type-2 diabetes) and behavioral health conditions (e.g., depression, somatization) [31–35]. Moreover, research has provided preliminary evidence in support of adapted behavioral activation interventions for treating depression among Latina/os and African Americans [36, 37].

The existing structures within senior housing facilities suggest that the use of these facilities can be effective for health interventions in older adults and may serve as a model to develop, test, and implement health promotion programs throughout the aging community [38]. This paper describes the rationale, study aims and objectives, and research design and methods of an ongoing 5-year, randomized clinical trial to test the efficacy of a home-based diabetes-modified behavioral activation treatment for low-income seniors with type 2 diabetes mellitus (HOME DM-BAT). The long-term goal of the project is to identify strategies to improve glycemic control and reduce diabetes complications and mortality in African Americans and Hispanics/Latinos with type 2 diabetes.

### Rationale

Persistent disparities based on race/ethnicity and socioeconomic status in healthcare are well documented in the USA [39–41]. Social determinants of health are key drivers of health disparities, especially in the elderly. T2DM is a chronic disease that is highly prevalent in the elderly, associated with significant racial/ethnic disparities, and impacted by social determinants of health [20, 21, 41, 42]. Elderly individuals with diabetes have high multimorbidity, complex treatment regimens, impaired functional status, and are often impacted by psychosocial determinants of health such as food insecurity, housing insecurity, competing needs, stress/coping, cognitive dysfunction, limited social and financial resources, and social isolation [16–19, 43, 44]. These factors create challenges for lifestyle and medical management [6]. Behavioral activation is a cognitive behavior therapy, originally developed to address depression, that has shown promise in individuals with chronic medical conditions, including T2DM [22–26]. The proposed study will test new strategies for improving clinical outcomes for T2DM in minority elders by addressing both diabetes-specific factors and social determinants of health that impede optimal health in this population.

### Study aim and objectives

The primary aim of the study is to test the efficacy of HOME DM-BAT on clinical outcomes (HbA1c, blood pressure, and LDL cholesterol). The secondary aim is to test the efficacy of HOME DM-BAT on behavioral outcomes (home blood glucose monitoring, diet, exercise, and medication adherence) and quality of life. The tertiary aim is to determine the cost-effectiveness of the HOME DM-BAT intervention for diabetes.

The primary outcome is HbA1c at 12 months post-randomization, while the secondary and tertiary outcomes are blood pressure control, low-density lipoprotein (LDL) cholesterol, behavioral outcomes (home blood glucose monitoring, diet, exercise, and medication adherence), quality of life, and cost-effectiveness.

## Methods

This 5-year study will evaluate the efficacy and cost-effectiveness of 8 sessions of in-home, culturally modified, manualized diabetes-modified, behavioral activation treatment (Home DM-BAT) delivered by trained diabetes nurse educators via telephone among low-income, ethnic minority seniors (age ≥65 years of age) with poorly controlled T2DM (HbA1c ≥8%), living in independent senior housing and community dwellings in Milwaukee using a randomized control trial design. Funding was awarded in April 2019 and has an anticipated end date of January 2024. Randomization will be at the individual level with blind outcome assessments. Assessments will occur at baseline, 3 months, 6 months, 9 months, and 12 months post-randomization.

### Location and settting

The study sites for this study are independent, subsidized senior housing, assisted living facilities, and community dwellings in low-income and high minority zip codes in Milwaukee, Wisconsin.

### Ethics and trial registration

The study is funded by grant R01DK118038 from the National Institute of Diabetes and Digestive and Kidney Diseases. The trial was approved by the Institutional Review Board (IRB) of the Medical College of Wisconsin in September 2019 (PRO00033789). The trial is registered (registration date, December 18, 2019) on the United States National Institutes of Health Clinical Trials Registry (ClinicalTrials.gov identifier# NCT04203147), available online at: http://clinicaltrials.gov/ct2/show/NCT04203147.

### Trial population and recruitment

A total of 200 African American and Hispanic/Latino participants will be randomized to one of two groups: (1) HOME DM-BAT intervention group, which consists of in-home, nurse telephone-delivered diabetes education, and behavioral activation and (2) general health education and supportive therapy group, with in-home, nurse telephone-delivered general health education.

Four complementary approaches will be used to identify eligible study subjects. The first method will consist of referrals from administrators and staff members at the senior housing sites. The PI will meet with the appropriate administrators at the senior sites to receive approval to conduct the study at the identified sites and recruit residents to participate in the study. In addition, the PI will provide a study overview, explain the study procedures, and discuss logistics for conducting the study at the senior housing sites. The administrators and staff members will be asked to recommend residents who they deem appropriate (i.e., poorly controlled glucose, motivated, history of participating in prior studies, and/or interest in research) to participate in the study. After receiving permission to approach those individuals, they will be invited to the baseline visit to assess eligibility. The second method will include the posting and distribution of recruitment flyers throughout the senior housing sites and to residents, respectively. The third approach will be to advertise the study via multiple strategies to community-dwelling elderly adults that are eligible for the study within the community. The fourth approach will consist of referrals from residents and community-dwelling elderly adults in response to recruitment flyers and advertisements. Those interested in participating in the study will be contacted for screening. If eligibility criteria are met, the baseline/enrollment visit will be scheduled.

Regardless of the recruitment pathway, research staff members obtain written informed consent, complete screening for eligibility, and assure that participants meet the criteria for inclusion and participation in the study. Inclusion criteria for study participants are as follows: (1) age ≥65 years of age, (2) self-identified as Black/African American or Hispanic, (3) clinical diagnosis of T2DM verified by an HbA1c ≥8% at the screening assessment, (4) able to communicate in English or Spanish, and (5) resident of independent, subsidized, assisted senior housing facility or community-dwelling elderly adults in the greater Milwaukee area and surrounding counties that have high African American/Hispanic populations. Exclusion criteria for study participants are as follows: (1) mental confusion at screening assessment suggesting significant dementia, (2) participation in other diabetes research, (3) alcohol or drug abuse/dependency at screening assessment, (4) active psychosis or acute mental disorder at screening assessment, and (5) life expectancy <12 months at screening assessment based on medical history and comorbidity screen used in prior studies.

The procedure and risks are explained to the patients and the consent form signed as per standard clinical practice. Participants who meet eligibility criteria then complete the remainder of the assessment battery. The study team will use reminder calls along with regular requests for updated contact information to promote participant retention and study completion.

### Confidentiality

Only members of the research team at MCW and the IRB will be allowed to handle participant health information. The study team has obtained a Certificate of Confidentiality to help protect the privacy of research participants. This certificate may be used to legally refuse to disclose information that may identify study participants in any federal, state, or local civil, criminal, administrative, legislative, or other proceedings. Study data is secured using a double lock system and shared servers are password and firewall-protected.

### Randomization

A permuted block randomization method will be used to assign subjects to one of the two intervention groups: (a) Home DM-BAT intervention and (b) Control (GHE/ST). Block size will be varied to minimize the likelihood that the blind will be broken. The randomization will be stratified by baseline HbA1c levels (8–10% vs. >10%). Using REDCap, RAs will collect eligibility information and enter the information into the study database via the secured study website [45]. Once eligibility is confirmed, the computer will generate the intervention assignment based on the pre-programmed randomization scheme. Study team members responsible for completing baseline and other follow-up assessments will be blinded to study group assignment. Study nurses delivering the intervention will be unblinded and will inform participants of their study group assignment during the baseline group visit. All participants who are randomized are entered into the study database and analyzed according to Consolidated Standards of Reporting Trials guidelines [46].

### Home-based diabetes-modified behavioral activation treatment intervention

A trained nurse educator will deliver the manualized Home DM-BAT intervention via telephone. Subjects will receive 8–weekly sessions of behavioral activation and monthly booster sessions from months 3 to 12. Home DM-BAT is extremely suitable to standard diabetes management approaches because it targets important diabetes management behaviors (medication taking, physical activity, health eating, and self-monitoring of glucose and BP). Moreover, Home DM-BAT specifically attempts to incorporate contingencies of environmental reinforcement for positive health behaviors, with a focus on medication adherence and other diabetes-specific healthy behaviors including physical activity, healthy eating, abstinence from smoking, and involvement in pleasurable activities that the patient chooses, such as spending time with friends, spirituality, or volunteer work. These supplemental activities are directed at improving overall emotional well-being. All intervention sessions will be delivered by telephone at times that do not interfere with previously scheduled activities. Each individual session will last 45 min and will include 15 min for a previously tested diabetes education/skills training intervention based on ADA guidelines, and 30 min for diabetes-tailored behavioral activation and to address social determinants of health issues.

### Usual care control group

Patients randomized to the control group will receive in-home, telephone-delivered 8–weekly sessions of combined general health education (GHE) and supportive therapy (ST) and monthly booster sessions from months 3 to 12. Given that this is an efficacy trial, the control group was designed to match the intervention group for both content and attention. The GHE component matches the diabetes education component of Home DM-BAT and ST component matches the behavioral activation component of Home DM-BAT. In addition, delivering GHE/ST to the control group will reduce attrition, maintain blinding, and maintain community trust. The control group will not receive diabetes education, address social determinants of health, or behavioral activation.

### Study instruments and data collection schedule

**Fig. 1:**
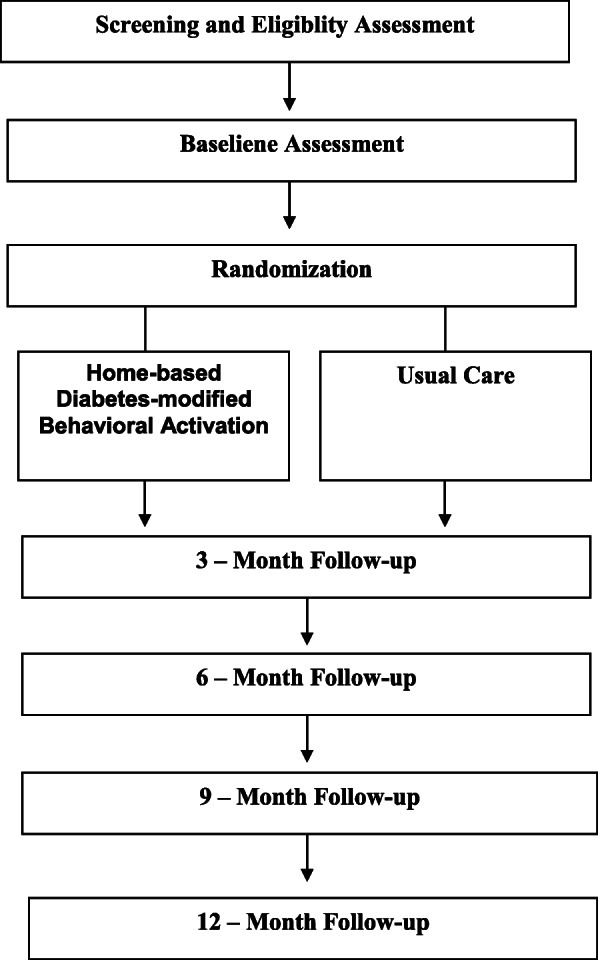
Design and study flow

**Fig. 2:**
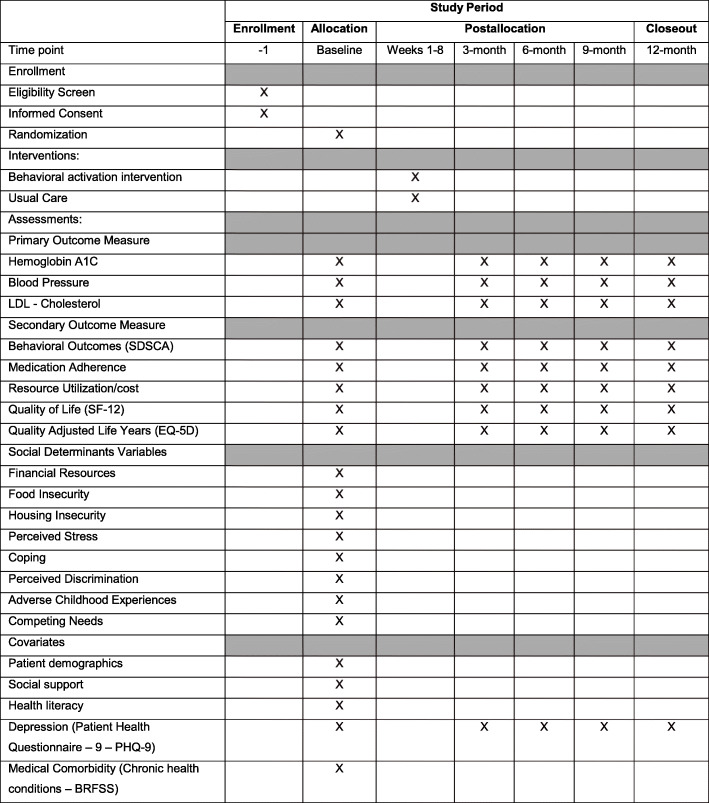
Schedule of enrollment, interventions, and assessments

See Figs. 1 and 2 and Table 1 for the study design and study flow, data collection schedule, and data collection measures and instruments, respectively.

**Table 1:** Data collection measures instruments

Measure	Method
**Primary outcomes**	
Hemoglobin A1C	Blood specimens will be obtained at baseline and at 3, 6, 9, and 12 months.
Blood pressure	Blood pressure readings will be obtained by study nurses using automated BP monitors (OMRON IntelliSenseTM HEM-907XL). The device will be programmed to take 3 readings at 2-min intervals and give an average of the 3 BP readings.
LDL - cholesterol	Blood specimens will be obtained at baseline and at 3, 6, 9, and 12 months.
**Secondary outcomes**	
Behavioral skills (SDSCA)	This will be assessed with the Summary of Diabetes Self-Care Activities (SDSCA) scale [47], a brief, validated self-report questionnaire of diabetes self-care.
Medication adherence	This will be measured with the 6-item validated self-report Brooks Medication Adherence Scale (BMAS) [48].
Resource utilization/cost	Previously validated questions that capture information on hospitalizations, physician visits, professional visits, and medications will be used.
Quality of life (SF-12)	The SF-12 [49] is a valid and reliable instrument to measure functional status and reproduces 90% of the variance in PCS-36 and MCS-36 scores.
Quality adjusted life years (EQ-5D)	The EQ-5D is a validated measure to assess health status developed by the EuroQol group and assess health status across 5 dimensions using 26 items [50].
**Social determinants**	
Financial resources	Financial resources will be measured using 7 items previously validated by Behavioral Risk Factor Surveillance System [51] regarding the social context in which a respondent lives.
Housing insecurity	Housing insecurity will be based on a series of 6 questions developed by Columbia University to measure the full spectrum of instability in housing over one year [52].
Food insecurity	6-item scale classifies household food security status and is valid for households with and without children [53]
Perceived stress	The perceived stress scale (PSS) is a 4-item scale that assesses the degree to which the respondent finds situations stressful [54].
Coping	Coping will be assessed using the 8-item emotional approach coping measure to allow evaluation of emotional processing and emotional expression [55].
Perceived discrimination	Previously validated items from the DISTANCE survey [56] will be used to capture perceived discrimination.
Adverse childhood experiences	The Adverse Childhood Experiences scale is a 10-item scale that assesses the degree to which the respondent experienced childhood maltreatment [57].
Competing needs	This will be assessed by validated questions that capture the impact of competing subsistence needs on healthcare [58].
**Covariates**	
Patient demographics	Previously validated items from the National Health Interview Survey will be used to capture demographic characteristics.
Social support	The Medical Outcomes Study (MOS) Social Support Survey [59] will be used to measure social support.
Health literacy	A 3-item literacy scale noting capacity to obtain, process, and understand basic health-related decisions will measure health literacy [60].
Depression (Patient Health Questionnaire – 9 – PHQ-9)	This will be assessed by the validated 9-item PHQ-9 [61].
Medical comorbidity (chronic health conditions—BRFSS)	This will be assessed using previously validated items from the Behavioral Risk Factor Surveillance System [62].

### Primary outcome measures

The primary outcomes are hemoglobin A1C (HbA1C), low-density lipoprotein (LDL) cholesterol, and blood pressure (BP) at 12-month follow-up assessed at baseline, 3 months, 6 months, 9 months, and 12 months post-randomization.

### Data management

The PI will be responsible for monitoring quality assurance and data integrity. Meetings with the PI, co-investigators, and study staff will take place weekly throughout the study. At each meeting, the team will discuss recruitment and accrual, protocol questions, retention, and data entry concerns. Enrollment numbers will be maintained and reported to the study team on an ongoing basis, along with information on reasons for exclusion, dates of completion of the study, and demographic summaries. All research staff will have training in human subject research, including data integrity and protection of confidentiality, and will follow standard procedures such as storage of hard copies of assessments and other data in locked file cabinets and separation of consent forms and identified master lists in separate locations. Study files will be maintained on an MCW server that provides nightly backup procedures and HIPPA compliant firewalls.

The MCW REDCap system will be used for data management. REDCap (Research Electronic Data Capture) is a secure, web-based application designed exclusively to support data capture for research studies initiated at Vanderbilt University (http://project-redcap.org/). REDCap provides (1) an intuitive interface for data entry (with data validation), (2) audit trails for tracking data manipulation and export procedures, (3) automated export procedures for seamless data downloads to common statistical packages (SPSS, SAS, Stata, R), (4) procedures for importing data from external sources, and (5) advanced features, such as branching logic and calculated fields.

### Safety monitoring

The safety monitoring plan includes an internal Data Safety Monitoring Committee (DSMC) and the institutional IRB. The purpose of the DSMC and IRB are to ensure the safety of participants and the validity and integrity of the data. Summaries of adverse events reports or patient safety concerns raised by the DSMC will be made to the IRB in the yearly progress unless the nature of a particular event is such that it bears reporting to IRB immediately.

The internal DSMC consists of the PI, biostatistician, co-investigators on the proposal, and the designated medical monitor. The functions of the DSMC include (1) provide scientific oversight, (2) review all adverse effects or complications related to the study, (3) monitor accrual, (4) review summary reports relating to compliance with protocol requirements, and (5) provide advice on resource allocation. The DSMC meets quarterly and as necessary by telephone. The recommendations of the DSMC will be reviewed, and the PI will take appropriate corrective actions as needed.

The IRB reviewed and approved the funded protocol, reviewed patient consent forms, and will ensure the protection of patient privacy and safety, and monitor the study on an ongoing basis. Adverse events will be reported to the IRB as they occur. Annual reports to the IRB will indicate accrual rate, adverse events, and new findings that may influence the continuation of the study and reports of the DSMC. Important protocol modifications will be made to the funding agency, IRB, and co-investigators.

### Sample size and power

For comparing the difference in outcome means for Home DM-BAT versus GHE/ST in an individually randomized design, measured at baseline and 3, 6, 9, and 12 months, with 80 participants per group, there will be 85% power to detect a *standardized* effect size ranging from 0.34 to 0.39 for the primary endpoint at 12 months [assuming levels of significance alpha=0.05 (two-tailed); 4 repeated measures for each participant; the correlation between repeated measures, rho, ranging from 0.5 to 0.7. Using the following pooled standard deviations from previous studies: 1.8 percentage point for HbA1c, 19.9 mmHg for SBP, 20.0 mmHg for LDL, and 12 points for SF-12, we will be able to detect raw differences (raw effect sizes) of approximately 0.61 to 0.70 percentage points in HbA1c, 6.77 to 7.77 mmHg in SBP, 6.8 to 7.8 mg/DL in LDL, and 4.08–4.68 points for SF-12 between the Home-DMBAT and GHE/ST groups. With 80% power, the standardized effect sizes that can be detected range from 0.32 to 0.36 for the given assumptions. The corresponding raw effect sizes are HbA1c (0.57 to 0.66 percentage points), SBP (6.33 to 7.27 mmHg), LDL (6.36 to 7.30 mg/DL), and SF-12 (3.82 to 4.38 points). To account for missing information in the ITT sample and the dilution effect of ITT analyses, we increase the sample size by 20% to achieve a final ITT sample size of 100 participants randomized 1:1 to each treatment group (total *N*=200 randomized participants).

### Data analysis

Baseline values for demographic, clinical, and other variables will be compared for imbalance across the two intervention groups (HOME DM-BAT and usual care). Primary analyses will use the ITT analysis set. These analyses will identify potential confounding variables to be used as covariates in subsequent analyses and will include race/ethnicity, gender, age, social support, health literacy, concurrent medical illness, depression, and motivation. Participants will not be discontinued from the study because of non-adherence and will remain in the study unless consent is withdrawn or there are concerns regarding patient safety.

The primary clinical outcome variables are HbA1c (glycemic control), blood pressure (SBP), and LDL-cholesterol; secondary outcome measures are behavioral [diet, exercise, medication adherence] and quality of life, measured at baseline and 3, 6, 9, and 12 months. The primary time point for evaluating intervention efficacy, as indicated by the magnitude and significance of differences in intervention outcome means is at 12 months (end of study). A longitudinal mixed-effects modeling procedure will be used as the general analytic framework for inferential analyses for individual and correlated multiple outcomes.

### Cost and cost-effectiveness analyses

The World Health Organization (WHO 2011) and the US Guidelines for cost-effectiveness analysis [Sanders 2016] will be used to estimate cost and cost-effectiveness from the provider, payer, and patient perspectives. From the provider and payer perspective, the total cost of the program per participant will be calculated using the resource cost method which includes personnel, overhead, supplies, and equipment costs necessary to provide Home DM-BAT. From the payer’s prospective, the cost of reimbursing the provider will also be calculated as cost. From the patient perspective, we will calculate the cost associated with behavior changes included in the study, such as changes in diet and physical activity, as well as estimating co-pays associated with the intervention. Effectiveness will be measured based on changes in HbA1c, quality of life (SF-12), and quality adjusted life years (QALYs). Data for cost estimates will be obtained through a combination of administrative records, clinical records, information collected as part of the patient interviews, and time-use data. QALYs will be based on the EQ-5D-3L administered to patients at baseline and follow-up, along with life expectancy tables from the CDC [63].

The patient-level analyses of cost and outcomes described above will be aggregated to produce overall cost-effectiveness measures. Using methods consistent with WHO and US Guidelines, we will conduct incremental cost-effectiveness analyses (CEAs) to assess the relative effects of the Home DM-BDAT intervention for 12 months of follow-up. Measures of effectiveness in the intervention period CEAs will be based on regression-adjusted observed differences across intervention and usual care groups in HbA1C level (primary) and quality of life (as measured by the SF-12). For the lifetime perspective, we will estimate gains in QALYs based on the EuroQoL [Schoenbaum 2001] for alternative trajectories of the disease [63], and health services utilization/cost, based on patient self-report of health services utilization and costs estimated from the Medical Expenditure Panel Survey (MEPS).

## Discussion

This study provides a unique opportunity to address existing gaps in the literature by testing a home-based, nurse telephone-delivered, diabetes, and behavioral activation intervention in elderly African Americans and Hispanic/Latinos. This study is novel in that it combines diabetes education with brief behavioral activation treatment and addresses social determinants of health (e.g., food insecurity, housing insecurity, competing needs, stress/coping) in minority seniors. In addition, Home DM-BAT directly addresses psychosocial factors, including complex treatment regimes, functional impairment, and social isolation [16–19, 43, 44], which may be grossly under-addressed in the management of diabetes in elderly populations. This is particularly important given that research strongly advocates for considering psychosocial issues and social context factors that can influence lifestyle behaviors in the treatment and management of diabetes, especially among racial/ethnic minority patients [33, 64]. Second, Home DM-BAT is a short-term, direct, and problem-solving-focused intervention, which researchers have argued is more congruent with racial/ethnic minority groups as these approaches are more congruent with their life circumstances that require immediate attention [65]. Moreover, directly in line with recommended guidelines, we have culturally adapted Home DM-BAT to increase participant engagement to promote positive clinical and quality of life outcomes [28, 29]. Finally, this study will provide comprehensive information on efficacy and cost-effectiveness, which can be combined with the experience of implementing the study at 30 different sites to guide future deployment and scalability across a broader population.

### Trial status

The study was funded in April 2019 and assigned protocol number 00033789. Study recruitment began in January 2020 but was suspended between March 16, 2020, and June 15, 2020, because of the COVID-19 pandemic. After being given clearance to restart recruitment efforts, the first group of patients was enrolled on July 10, 2020, and participants are actively being recruited. As of June 2, 2021, 72 of 200 total participants have been randomized. Recruitment is expected to be completed by January 2023.

## Data Availability

LEE will have access to the final trial dataset and study-related materials.

## References

[1] US Department of Health and Human Services Office of Minority Health (2019). Diabetes and African Americans.

[2] Centers for Disease Control and Prevention (2019). Hispanic/Latino Americans and type 2 diabetes.

[3] American Diabetes Association (2018). Statistics about diabetes: overall numbers.

[4] Center for Disease Control and Prevention (CDC) (2017). National Diabetes Statistics Report, 2017.

[5] Agency for Healthcare Research and Quality (AHRQ) (2001). Diabetes disparities among racial and ethnic minorities. AHRQ Publication No. 02-P007.

[6] Kirkman MS, Briscoe VJ, Clark N, Florez H, Haas LB, Halter JB, Huang ES, Korytkowski MT, Munshi MN, Odegard PS, Pratley RE, Swift CS (2012). Diabetes in older adults. Diabetes Care..

[7] Federal Interagency Forum on Aging-Related Statistics (2016). Older Americans 2016: key indicators of well-being. Federal Interagency Forum on Aging-Related Statistics.

[8] Willey JZ, Moon YP, Kahn E, Rodriguez CJ, Rundek T, Cheung K, Sacco RL, Elkind MS (2014). Population attributable risks of hypertension and diabetes for cardiovascular disease and stroke in the northern Manhattan study. J Am Heart Assoc..

[9] Villa VM, Wallace SP, Bagdasaryan S, Aranda MP (2012). Hispanic baby boomers: health inequities likely to persist in old age. The Gerontologist.

[10] Moody-Ayers SY, Mehta KM, Lindquist K, Sands L, Covinsky KE (2005). Black-White disparities in functional decline in older persons: the role of cognitive function. J Gerontology.

[11] Anderson NB, Bulatao RA, Cohen B (2004). Critical perspectives on racial and ethnic differences in health in late life.

[12] Avendano M, Kawachi I, VanLenthe F, Boshuizen HC, Mackenbach JP, denBos V, Fay ME, Berkman LF (2006). Socioeconomic status and stroke incidence in the US elderly: the role of risk factors in the EPESE study. Stroke.

[13] Marmot M (2005). Social determinants of health inequalities. Lancet.

[14] Williams DR, Mohammed SA (2009). Discrimination and racial disparities in health: evidence and needed research. Journal of Behavioral Medicine.

[15] Pascoe EA, Richman LS (2009). Perceived discrimination and health: a meta-analytic review. Psychological Bulletin.

[16] Young-Hyman D, de Groot M, Hill-Briggs F, Gonzalez JS, Hood K, Peyrot M (2016). Psychosocial care for people with diabetes: a position statement of the American Diabetes Association. Diabetes Care.

[17] Young-Hyman D, de Groot M, Hill-Briggs F, Gonzalez JS, Hood K, Peyrot M (2016). Psychosocial care for people with diabetes: a position statement of the American Diabetes Association. Diabetes Care.

[18] Tomaka J, Thompson S, Palacios R (2006). The relation of social isolation, loneliness, and social support to disease outcomes among the elderly. Journal of Aging and Health.

[19] Tomaka J, Thompson S, Palacios R (2006). The relation of social isolation, loneliness, and social support to disease outcomes among the elderly. J Aging Health.

[20] Walker RJ, Gebregziabher M, Martin-Harris B, Egede LE (2015). Quantifying direct effects of social determinants of health on glycemic control in adults with type 2 diabetes. Diabetes Technology and Therapeutcis.

[21] Walker RJ, Smalls BL, Egede LE (2015). Social determinants of health in adults with type 2 diabetes--contribution of mutable and immutable factors. Diabetes Res Clin Pract..

[22] Lejuez CW, Hopko DR, Hopko SD (2001). A brief behavioral activation treatment for depression. Behavior Modification.

[23] Lejuez CW, Hopko DR, Acierno R, Daughters SB, Pagoto SL (2011). Ten year revision of the brief behavioral activation treatment for depression (BATD): revised treatment manual (BATD-R). Behavioral Modification.

[24] Schneider KL, Panza E, Handschin B, Ma Y, Busch A, Waring ME, Appelhans BM, Whited MC, Keeney J, Kern D, Blendea M, Ockene I, Pagoto SL (2016). Feasibility of pairing behvaioral activation with exercise for women with type 2 diabetes and depression: the get it study pilot randomized controlled trial. Behavioral Therapy.

[25] Kaltman S, de Mendoza AH, Serano A, Gonzales FA (2016). A mental health intervention strategy for low-income, trauma-exposed Latina immigrants in primary care. A preliminary study. American Journal of Orthopsychiatry.

[26] Weiss DM, Casten RJ, Leiby BE, Hark LA, Murchison AP, Johnson D, Stratford S, Henderer J, Rovner BW, Haller JA (2015). Effect of behavioral intervention on dilated fundus examination rates in older African American individuals with diabetes mellitus: a randomized clinical trial. JAMA Ophthalmol..

[27] Hopko DR, Lejuez CW, Ruggiero KJ, Eifert GH (2003). Contemporary behavioral activation treatments for depression: procedures, principles, and progress. Clinical Psychology Review.

[28] Barrera M, Castro FG, Strycker LA, Toobert DJ (2013). Cultural adaptations of behavioral health interventions: a progress report. Journal of Consulting and Clinical Psychology.

[29] Smith PB (2011). Communication styles as dimensions of national culture. Journal of Cross-Cultural Psychology.

[30] Wingood GM, DiClemente RJ (2008). The ADAPT-ITT Model: a novel method of adapting evidence-based HIV interventions. Journal of Acquired Immune Deficiency Syndrome.

[31] DiClemente RJ, Wingood GM, Harrington KF, Lang DL, Davies SL, Hook EW, Oh KM (2004). Efficacy of an HIV prevention intervention for African American adolescent females: a randomized controlled trial. Journal of the American Medical Association.

[32] Interian A, Allen LA, Gara MA, Escobar JI (2008). A pilot study of culturally adapted cognitive behavior therapy for Hispanics with major depression. Cognitive and Behavioral Practice.

[33] Two Feathers J, Kieffer EC, Palmisano G, Anderson M, Sinco B, Janz N (2005). Racial and Ethnic Approaches to Community Health (REACH) Detroit partnership: improving diabetes-related outcomes among African American and Latino adults. American Journal of Public Health.

[34] Vincent D, Pasvogel A, Barrera L (2007). A feasibility study of a culturally tailored diabetes intervention for Mexican Americans. Biological research for nursing.

[35] Juckett G (2013). Caring for Latino patients. American Family Physician.

[36] Kanter JW, Santiago-Rivera AL, Rusch LC, Bushch AM, West P (2010). Initial outcomes of a culturally adapted behavioral activation for Latinas diagnosed with depression at a community clinic. Behavior Modification.

[37] Saulsberry A, Corden ME, Taylor-Crawford K, Crawford TJ, Johnson M, Froemel J (2013). Chicago urban resiliency building (CURB): an internet-based depression-prevention intervention for urban African-American and Latino adolescents. Journal of Child and Family Studies.

[38] Speer EM, Reddy S, Lommel TS, Fischer JG, Heather S, Park S, Johnson MA (2008). Diabetes self-management behaviors and A1c improved following a community-based intervention in older adults in Georgia senior centers. J Nutr Elder..

[39] Institute of Medicine (US) Committee on Understanding and Eliminating Racial and Ethnic Disparities in Health Care. Unequal Treatment: Confronting Racial and Ethnic Disparities in Health Care. Smedley BD, Stith AY, Nelson AR, editors. Washington (DC): National Academies Press (US); 2003. 25032386

[40] Agency for Healthcare Research and Quality. 2015 National Healthcare Quality and Disparities Report. Rockville: US Department of Health and Human Services; 2015.

[41] Williams JS, Walker RJ, Smalls BL, Hill R, Egede LE (2016). Patient-centered care, glycemic control, diabetes self-care, and quality of life in adults with type 2 diabetes. Diabetes Technol Ther..

[42] Walker RJ, Strom Williams J, Egede LE (2016). Influence of race, ethnicity and social determinants of health on diabetes outcomes. Am J Med Sci..

[43] Billimek J, Sorkin DH (2012). Self-reported neighborhood safety and nonadherence to treatment regimens among patients with type 2 diabetes. J Gen Intern Med.

[44] Nicklett EJ, Heisler MEM, Spencer MS, Rosland AM (2013). Direct social support and long-term health among middle-aged and older adults with type 2 diabetes mellitus. The Journals of Gerontology Series B: Psychological Sciences and Social Sciences.

[45] Harris PA, Taylor R, Thielke R, Payne J, Gonzalez N, Conde JG (2009). Research electronic data capture (REDCap) – a metadata-driven methodology and workflow process for providing translational research informatics support. Journal of Biomedical Informatics.

[46] Altman DG, Schulz KF, Moher D, Egger M, Davidoff F, Elbourne D, Gotzsche PC, Lang T, CONSORT Group (Consolidated Standards of Reporting Trials). (2001). The revised CONSORT statement for reporting randomized trials: explanation and elaboration. Annals of Internal Medicine*,* 134: 663 – 694. 10.7326/0003-4819-134-8-200104170-00012 11304107

[47] Toobert DJ, Hampson SE, Glasgow RE. The summary of diabetes self-care activities measure: results from 7 studies and a revised scale. Diabetes Care. 2000;23(7):943–50. 10.2337/diacare.23.7.943 10895844

[48] Brooks CM, Richards JM, Kohler CL, Soong SJ, Martin B, Windsor RA, Bailey WC. Assessing adherence to asthma medication and inhaler regimens: a psychometric analysis of adult self-report scales. Med Care. 1994;32(3):298–307. 10.1097/00005650-199403000-00008 8145604

[49] Ware JE, Kosinski M, and Keller SD. A 12-Item Short-Form Health Survey: Construction of scales and preliminary tests of reliability and validity. Medical Care. 1996;34(3):220–33. 10.1097/00005650-199603000-00003 8628042

[50] Herdman M, Gudex C, Lloyd A, Janssen M, Kind P, Parkin D, Bonsel G, Badia X. Development and preliminary testing of the new five-level version of EQ-5D (EQ-5D-5L). Qual Life Res. 2011;20(10):1727–36. 10.1007/s11136-011-9903-x PMC3220807 21479777

[51] Centers for Disease Control and Prevention. Behavioral Risk Factor Surveillance System. Atlanta: National Center for Chronic Disease Prevention and Health Promotion, Division of Population Health; 2014.

[52] Curtis MA, Geller AB. Housing insecurity among urban fathers. New York: Columbia University Libraries; 2010.

[53] Bickel G, Nord M, Price C, Hamilton W, Cook J. Guide to Measuring Household Food Security. Alexandria: US Department of Agriculture; 2000.

[54] Cohen S. Perceived stress in a probability sample of the United States. In S. Spacapan & S. Oskamp (Eds.). The social psychology of health. Sage Publications; 1988. pp. 31–67.

[55] Stanton AL, Kirk SB, Cameron CL, Danoff-Burg S. Coping through emotional approach: scale construction and validation. J Pers Soc Psychol. 2000;78(6):1150–69. 10.1037//0022-3514.78.6.1150 10870915

[56] Moffet HH, Adler N, Schillinger D, Ahmed AT, Laraia B, Selby JV, Neugebauer R, Liu JY, Parker MM, Warton M, Karter AJ. Cohort Profile: The Diabetes Study of Northern California (DISTANCE)—objectives and design of a survey follow-up study of social health disparities in a managed care population. Int J Epidemiol. 2009;38(1):38–47. 10.1093/ije/dyn040 PMC2635421 18326513

[57] Felitti VJ, Anda RF, Nordenberg D, Williamson DF, Spitz AM, Edwards V, Koss MP, Marks JS. Relationship of childhood abuse and household dysfunction to many of the leading causes of death in adults. The Adverse Childhood Experiences (ACE) Study. Am J Prev Med. 1998;14(4):245–58. 10.1016/s0749-3797(98)00017-8 9635069

[58] Cunningham WE, Andersen RM, Katz MH, Stein MD, Turner BJ, Crystal S, Zierler S, Kuromiya K, Morton SC, St. Clair P, Bozzette SA, Shapiro MF. The impact of competing subsistence needs and barriers on access to medical care for persons with human immunodeficiency virus receiving care in the United States. Medical Care. 1999;37(12):1270–81. 10.1097/00005650-199912000-00010 10599608

[59] Sherbourne CD, Stewart AL. The MOS Social Support Survey. Soc Sci Med. 1991;32:705–14. 10.1016/0277-9536(91)90150-b 2035047

[60] Chew LD, Griffin JM, Partin MR, Noorbaloochi S, Grill JP, Snyder A, Bradley KA, Nugent SM, Baines AD, Vanryn M. Validation of screening questions for limited health literacy in a large VA outpatient population. J Gen Intern Med. 2008;23(5):561–6. 10.1007/s11606-008-0520-5 PMC2324160 18335281

[61] Kroenke K, Spitzer RL, Williams JB. The PHQ-9: validity of a brief depression severity measure. J Gen Intern Med. 2001;16(9):606–13. 10.1046/j.1525-1497.2001.016009606.x PMC1495268 11556941

[62] Centers for Disease Control and Prevention. Behavioral Risk Factor Surveillance System. National Center for Chronic Disease Prevention and Health Promotion. Atlanta: Division of Population Health; 2014.

[63] CDC Diabetes Cost-Effectiveness Group (2002). Cost-effectiveness of intensive glycemic control, intensified hypertension control, and serum cholesterol level reduction for type 2 diabetes. JAMA.

[64] Samuel-Hodge CD, Headen SW, Skelly AH, Ingram AF, Keyserling TC, Jackson EJ, Ammerman AS, Elasy TA (2000). Influences on day-to-day self-management of type 2 diabetes among African-American women: spirituality, the multi-caregiver role, and other social context factors. Diabetes Care.

[65] Organista KC, Munoz RF. Cognitive behavioral therapy with Latinos. Cogn Behav Pract. 1996;3:255–70.

